# Feasibility of Combining Biomolecular Conformational Sampling Techniques for Molecular Dynamics Simulation

**DOI:** 10.1002/jcc.70192

**Published:** 2025-07-30

**Authors:** Jinzen Ikebe, Hidetoshi Kono

**Affiliations:** ^1^ Computational Omics Research Team Artificial Intelligence Research Center, National Institute of Advanced Industrial Science and Technology (AIST), AIST Tokyo Waterfront BIO‐IT Research Building Tokyo Japan; ^2^ Molecular Modeling and Simulation Team Institute for Quantum Life Science (iQLS), National Institutes for Quantum Science and Technology (QST) Chiba Japan; ^3^ Center of Quantum Life Science for Structural Therapeutics (cQUEST), Chiba University Chiba Japan

**Keywords:** conformational sampling, cutoff based method, electrostatic interaction, generalized ensemble method, molecular dynamics simulation

## Abstract

We assess the feasibility of combining two advanced molecular dynamics techniques for efficient biomolecular conformational sampling: the generalized ensemble method for enhancing conformational sampling in partial systems (GEPS), such as ALSD and REST2, which dynamically modulate atomic charges in selected regions, and the zero‐multipole summation method (ZMM), which efficiently computes electrostatic interactions assuming local electrostatic neutrality. To address whether charge variation in GEPS violates the fundamental assumption of ZMM, we compared conformational ensembles obtained using GEPS combined with either ZMM or a conventional electrostatic calculation method. Our results demonstrate that GEPS and ZMM can be effectively combined without introducing systematic bias. Additionally, we identified a potential limitation of ZMM: in highly polarized systems, it may fail to capture long‐range electrostatic repulsion, potentially leading to artifacts. These findings support the practical use of GEPS with ZMM for conformational sampling; however, caution is warranted when applying ZMM to systems with highly delocalized electrostatics.

## Graphical abstract

We assess the feasibility of combining GEPS, generalized ensemble method for enhancing conformational sampling in partial systems, with zero‐multipole summation method (ZMM), which assumes local electrostatic neutrality for efficient electrostatic energy calculations. Since GEPS alters atomic charges, its compatibility with ZMM was uncertain. Our study confirms that conformational ensembles, free energy landscapes, and local electrostatic environments remain consistent with a traditional electrostatic calculation method, demonstrating the combined approach's reliability for accurate and efficient biomolecular simulations.

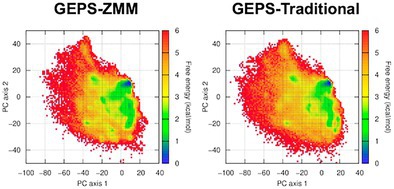

## 1 Introduction

Molecular dynamics (MD) simulation [1, 2, 3, 4] is a powerful tool that studies the motion of biomolecules. The ability to provide detailed information at atomic‐level spatial resolution and femtosecond‐level temporal resolution helps us to reveal processes such as conformational changes in biomolecules, complex formation with other molecules, and protein folding. The so‐called generalized ensemble (GE) [5, 6, 7, 8] method can sample various possible biomolecular conformations in equilibrium more quickly and efficiently than conventional simulations. Biomolecules often adopt specific, most stable structures (native structures) to perform their functions. However, highly long simulations are generally required to identify these native structures among numerous possible structures [9]. Multi‐degree‐of‐freedom systems, such as biomolecules immersed in the solvent molecules, must traverse a complicated, rugged energy landscape with multiple energy minima (metastable structures) and energy barriers (transition structures) to reach the native structures. The GE method mitigates trapping in local energy minima by performing conformational sampling based on artificial ensembles rather than strictly following the molecular motion. This approach enables faster and more efficient conformational searches compared to conventional simulation. Furthermore, the GE method allows the reconstruction of canonical ensembles from the resulting artificial ensembles using a reweighting scheme [10, 11, 12, 13], making it possible to evaluate various physical quantities with high statistical accuracy.

Multicanonical MD (McMD)‐type GE overcomes energy barriers by introducing artificial potential energy. McMD [7, 14, 15], one of the umbrella sampling [16] methods, achieves a random walk in the potential energy space by adding multicanonical potentials estimated through trial simulations. This random walk enables the simultaneous exploration of stable structures in low‐energy regions and the promotion of conformational changes in high‐energy regions. McMD has been successfully applied to protein folding [17, 18, 19] and molecular docking [20, 21] studies. Variants of McMD have also been developed to improve the efficiency in conformational searches further [22, 23, 24, 25, 26, 27, 28, 29, 30, 31, 32, 33]. The mypresto/omegagene [34] and j_presto [35] MD program suites offer several McMD‐type GEs. Another typical MD approach with the GE method is replica exchange MD (REMD)‐type GE, which overcomes energy barriers by simultaneously running multiple copies (replicas) of MD simulations under slightly different conditions and exchanging adjacent replicas under certain conditions. The original REMD [36, 37] simultaneously runs replicas of canonical MD simulations, each at a different temperature, and exchanges temperature parameters with probabilities determined by the Metropolis criterion. This approach facilitates a random walk across a wide temperature range from low to high temperatures. Since then, various REMD variants [38, 39, 40, 41, 42, 43] have been developed that exchange parameters other than temperature. Unlike McMD‐type GE, REMD‐type GE does not require trial simulations, making handling easier. Consequently, it has been implemented into general‐purpose MD software such as GROMACS [44, 45], AMBER [46], NAMD [47], CHARMM [48], and GENESIS [49]. In REMD‐type GEs, on the other hand, the computable system size is limited by the amount of available computing resources because the number of replicas simultaneously executed should be in proportion to the square root of the system size [37, 39, 50, 51].

Conventional GE methods require random walks across a wide range of potential energies or temperatures of the entire system. However, in biomolecular simulations, the total energy mainly comes from solvent‐related interactions, and the contribution of the solute‐related energy to it is quite small. Therefore, methods that effectively enhance the sampling of solute conformations, such as selectively enhanced McMD [52, 53], replica exchange with solute tempering (REST) [54], and its improved version (REST2) [55] were developed. Subsequently, the selection of enhanced regions was further refined, leading to the development of new methods such as McMD‐type GEs (partial McMD [56] and ALSD [57]) and REMD‐type GEs (replica exchange with flexible tempering (REFT) [58] and generalized REST (gREST) [59]). These advanced methods enable selective enhancement of conformational sampling in arbitrary regions, not only within solutes but also in any part of the system, including specific energy terms (e.g., torsion angle energy, electrostatic energy, van der Waals (vdW) energy, etc.). This paper collectively refers to these techniques as generalized ensemble methods for enhancing conformational sampling in partial systems (GEPS). GEPSs have been successfully applied to studying conformations of structurally fluctuating loop regions in proteins [58, 60, 61, 62] and docking pose searches of ligands in protein complexes [63, 64, 65, 66, 67]. Conventional GEs, which promote conformational changes throughout the systems, often end up disrupting stable structures we want to keep intact. However, GEPSs can maintain conformational changes other than the selected regions to the extent of conventional MD.

While GEPSs generally improve sampling efficiency, not all of them consistently outperform conventional GEs. For example, Huang et al. [50] reported that REST is not always more efficient than REMD. In contrast, Wang et al. [55] showed that REST2 offers a significant improvement in sampling efficiency compared to REST. This discrepancy highlights the importance of method‐specific refinements within GEPSs. Ikebe et al. [22, 57] further elucidated factors underlying these performance differences, emphasizing the critical role of parameter variability in achieving efficient conformational sampling for GEPSs. GEPSs can be broadly categorized into two types: parameter‐variable GEPSs, such as ALSD and REST2, and non‐parameter‐variable GEPSs, including partial McMD and the original REST. Parameter‐variable GEPSs employ a methodology in which atomic parameters, such as charges for electrostatic energy, Lennard‐Jones potential depths for vdW energy, and spring constants for covalent bonded energies, are treated as if they dynamically change during simulation. This approach enables molecules in selected regions to behave like random chains at high temperatures, facilitating the exploration of vast conformational space. By contrast, non‐parameter‐variable GEPSs often face challenges in high energy areas, where molecules may become confined to limited structures such as fully elongated or aggregated structures (for more details, see ALSD papers [22, 57]). This problem becomes particularly pronounced in larger, highly polarized systems, though it is less obvious in small systems such as alanine dipeptide, which is used as a test system to validate many GE methods. Moreover, the choice of energy terms for promoting conformational changes significantly influences sampling efficiency. Torsion angle, electrostatic, and vdW energies are critical to global conformational changes. However, GEPSs that select either electrostatic or vdW energy terms were less effective in conformational searches [22]. In this paper, the term “GEPSs” specifically refers to parameter‐variable GEPSs that promote energy changes across at least three energy terms mentioned above, and the GEPS is one of the two core techniques employed in this study.

The second simulation technique employed in this paper is the zero‐multipole summation method (ZMM) [68, 69, 70], developed by Fukuda et al. as an efficient approach to calculate electrostatic energy. Unlike covalent bond energies, the computation of electrostatic and vdW energies between all atom pairs is a time‐consuming task occupying most of the simulation cost: if they are calculated rigorously, the computational cost scales as *O*(*N* ^2^). For vdW interactions, which decay rapidly and become negligible beyond approximately 10 Å, the cutoff calculation is typically applied to ignore interactions between atom pairs separated by distances exceeding the cutoff radius. In contrast, electrostatic interactions act at farther distances, so applying the cutoff calculation introduces non‐negligible errors compared to exact calculation [71, 72, 73, 74]. Therefore, it is essential to develop an efficient method for calculating electrostatic energy that achieves a balance between computational accuracy and efficiency.

Currently, the Ewald‐based methods are the most commonly used for calculating electrostatic energy in many MD simulation programs [75, 76]. They assume periodic boundary conditions with infinitely replicated copies (image cells) of the target molecular system. By exploiting periodicity, electrostatic energy can be expanded into a Fourier series to calculate contributions from the image cells at infinity. Since the Fourier series expansion is computationally expensive, particle‐particle particle‐mesh (PPPM or P3M) [77, 78] particle‐mesh Ewald (PME) [79], smooth PME (SPME) [80], and Gaussian‐split Ewald [81] methods are used to accelerate the process using fast Fourier transforms (FFT) reducing computational complexity to *O*(*N*log*N*). Another effective technique is multipole expansion methods, such as cell multipole method (CMM) [82], particle‐particle particle‐cell method (PPPC) [83], and fast multiple method (FMM) [84, 85]. In these methods, the system is divided into a lattice of cells. Interactions from atoms in neighboring cells are calculated rigorously, while interactions between atoms in distant cells are approximated using multiple expansions. Unlike Ewald‐based methods, these approaches have the advantage of being applicable to non‐periodic boundary conditions without the need for the computationally expensive FFT. As a result, the computational complexity is reduced to *O*(*N*log*N*) for CMM and PPPC, and *O*(*N*) for FMM, while maintaining accuracy by calculating nearby interactions with significant effects and approximately evaluating distant interactions with relatively minor impact.

ZMM method [68, 69, 70] is also one of the non‐Ewald methods [86, 87] and is based on the cutoff method, using the physical properties of biomolecular systems to achieve accurate electrostatic energy calculations, rather than approximating distant interactions. In aggregated systems of charged particles, such as biomolecular systems, negatively charged particles tend to be distributed around positive ones, and vice versa. In other words, atomic populations in biomolecular systems are arranged in a way that electrostatic interactions largely cancel out, and the probability of a highly biased charge distribution in a particular region is extremely low. Conventional cutoff calculations often artificially generate physically undesirable nonzero charge states because the space is truncated within a finite small radius. Wolf et al. found that significant errors in electrostatic energy in the cutoff calculations were due to the nonzero charge states. They developed the so‐called Wolf method [88, 89], which conceptually prevents the occurrence of nonzero charge states by assuming that any imbalance within the cutoff sphere is neutralized by a virtual distribution of charges on the surface of the sphere. This approach ensures electrostatic neutrality even when the truncated region itself does not satisfy the neutrality and improves the accuracy of electrostatic energy calculations. Fukuda et al. developed a zero‐dipole summation method (ZDM) [90, 91], which extends this concept by neutralizing not only nonzero charge states but also nonzero dipole states. They demonstrated that the energy calculated using ZDM agrees well with PME's for a water molecule system. They further extended ZDM to ZMM, which neutralizes higher‐order multipoles, and found that neutralization with higher orders leads to more accurate electrostatic energy calculation.

The advantages of ZMM are its high scalability in *O*(*N*) and its high performance in parallel computing. Recent advancements of computer performance enable simulations of larger systems and for longer durations. For such large‐scale simulations, multi‐CPU parallel computing with MPI and OpenMP or GPU parallel computing with CUDA is essential. These approaches often employ spatial domain decomposition [92], which divides the physical geometry into boxes assigned to each processor. However, calculating long‐range electrostatic energies requires accessing data stored in the memory of distant processors. In modern hardware, inter‐processor communication presents a significant bottleneck because it is much more costly than computation. In this context, ZMM does offer a distinct advantage because the electrostatic energy calculation is formulated as a simple pairwise sum over atoms within the cutoff radius, significantly reducing inter‐processor communication compared to methods relying on FFT or multipole expansions. This characteristic makes ZMM highly scalable in parallel computing, as demonstrated in detail by Sakuraba et al. [70]. Moreover, the cutoff‐based ZMM is applicable regardless of boundary conditions, unlike Ewald‐based methods—this flexibility of ZMM positions it as a promising alternative for electrostatic energy calculations in modern MD simulations.

GEPS combined with ZMM is a promising approach to advance conformational exploration of biomolecules as they are recognized for its excellent scalability in parallel computations. This combination has already been applied and examined for studying intrinsically disordered regions [60, 61, 62, 64] and searching for substrate docking poses in enzyme pockets [63, 93]. However, whether it is proper to combine GEPSs with ZMM arises: in GEPSs, atomic charges change during simulations, but ZMM assumes local electrostatic neutrality around atoms. When employing GEPS, the electrostatic properties around atoms may deviate from those in conventional MD simulations, potentially violating electrostatic neutrality. That indicates that GEPS with conventional electrostatic interaction calculation methods results in reflecting the non‐neutral states. In contrast, combining GEPS with ZMM would enforce calculations under the assumption of electrostatic neutrality, even if the actual electrostatic state deviates from neutrality. This fundamental methodological difference could raise concerns about discrepancies between calculated charge distributions and conformational ensembles. Despite these potential implications, no systematic investigation has been conducted to verify whether such issues arise.

Ideally, it would be desirable to mathematically prove that the conformational ensembles and statistical physical quantities obtained under the combination of GEPS and ZMM are equivalent to those obtained with conventional electrostatic interaction methods. Such a proof would require demonstrating that even under conditions of dynamically changing atomic charges, the ZMM assumption—namely, that electrostatic interactions from locally neutral atomic groups within a cutoff radius can approximate exact electrostatic interactions—still holds. However, since this assumption was introduced as a foundational premise in the development of ZMM, rather than as something that can be derived or proven mathematically, providing rigorous justification is inherently challenging. Therefore, as an alternative to mathematical proof, this study aims to provide empirical evidence supporting the equivalence of the two approaches by comparing conformational ensembles and statistical quantities.

To investigate the abovementioned issues, we conducted conformational searches using GEPS in combination with either ZMM or a conventional electrostatic energy calculation method for three systems: relatively neutral proteins, chignolin and trp‐cage, which possess different secondary structures, and a highly positively charged poly‐lysine heptapeptide. These proteins were chosen as target systems due to their small size, making them ideal for efficiently exploring precise conformational landscapes within a practical computational time frame. Our results reveal two key findings. First, GEPS and ZMM can be effectively combined without causing systematic bias in conformational sampling. Second, we identified a limitation inherent to ZMM: when a small cutoff radius is used, ZMM may underestimate long‐range electrostatic repulsion. This issue appears to be particularly significant in highly polarized systems, such as poly‐lysine, and can influence the resulting conformational ensembles. However, we found that increasing the cutoff radius reduces these discrepancies and brings ZMM results closer to those obtained with conventional methods.

## 2 Methods

### 2.1 Adaptive Lambda Square Dynamics (ALSD) Simulation

This study used ALSD as one of the GEPSs. Here, we briefly explain ALSD (see the reference [57] for the details). ALSD realizes an effective sampling by modifying the energy of the simulated system during the simulation. In a conventional MD simulation, a potential energy is represented as

$$E=E_{\mathrm{ele}}+E_{\mathrm{vdW}}+E_{\text{bonded}}$$

(1) $$=\frac{1}{4\pi \varepsilon_{0}}\sum_{i<j}\frac{q_{i}q_{j}}{r_{\mathrm{ij}}}+\sum_{i<j}\sqrt{\varepsilon_{i}}\sqrt{\varepsilon_{j}}\left[{\left(\frac{\sigma_{\mathrm{ij}}}{r_{\mathrm{ij}}}\right)}^{12}-{\left(\frac{\sigma_{\mathrm{ij}}}{r_{\mathrm{ij}}}\right)}^{6}\right]+\sum \frac{1}{2}k_{\mathrm{ij}}^{b}f\left(x\right)$$

The *E*_ele_ is the electrostatic energy term, where *q*_*i*_ is the point charge of atom *i*, *ε*_0_ is the dielectric constant, and *r*_*ij*_ is the distance between atom *i* and *j*. The *E*_vdW_ denotes the van der Waals (vdW) energy term, where *σ*_*ij*_ is the average vdW radii of atoms *i* and *j*, and the product $\sqrt{\varepsilon_{i}}\sqrt{\varepsilon_{j}}$ is the depth of the Lennard‐Jones potential well for a pair of atoms *i* and *j*, expressed by Lorentz‐Berthelot combining rules. The *E*_bonded_ is the energy term related to covalent bonds such as bond, angle, torsion angle, and improper torsion angle, where $k_{\mathrm{ij}}^{b}$ is a spring constant for a pair of atoms, *i* and *j*, and *f*(*x*) is a function of a parameter × corresponding to the bond‐length, bond‐angle, torsion‐angle, or improper torsion angle. In ALSD simulation, the system is divided into two regions *A* and *B*: the region *A* (solute molecules in this work) where the conformational changes in the region *A* are enhanced, and the region *B* (ions and water molecules) where the thermal fluctuations of molecules are maintained as in the canonical simulation. In ALSD, the potential energy *E*_ALSD_ is modified and scaled by a scaling factor, *λ* and is given as

(2) $$\begin{array}{c} E_{\text{ALSD}}=\frac{1}{4\pi \varepsilon_{0}}\sum_{i<j}\frac{\left(\lambda_{i}q_{i}\right)\left(\lambda_{j}q_{j}\right)}{r_{\mathrm{ij}}}\\ \;+\sum_{i<j}\left(\lambda_{i}\sqrt{\varepsilon_{i}}\right)\left(\lambda_{j}\sqrt{\varepsilon_{j}}\right)\left[{\left(\frac{\sigma_{\mathrm{ij}}}{r_{\mathrm{ij}}}\right)}^{12}-{\left(\frac{\sigma_{\mathrm{ij}}}{r_{\mathrm{ij}}}\right)}^{6}\right]\\ \;+\sum \frac{1}{2}\left(\lambda_{i}\sqrt{k_{\mathrm{ij}}^{b}}\right)\left(\lambda_{j}\sqrt{k_{\mathrm{ij}}^{b}}\right)f\left(x\right) \end{array}$$

where $\lambda_{i}=\lambda$ when atom *i* belongs to the region *A*, otherwise $\lambda_{i}=1$. Note that parameters such as *q*_*i*_, $\sqrt{\varepsilon_{i}}$, and $\sqrt{k_{\mathrm{ij}}^{b}}$ for atoms in the region *A* are scaled by the *λ*. In this study, the energy scaling was applied to the electrostatic, vdW, and torsion angle energies (except for those around peptide bonds). In contrast, the energy terms for bond length, bond angle, torsion angle around peptide bonds, and improper torsion angle were not scaled, i.e., were treated as in the canonical simulation.

During ALSD simulation, the scaling factor *λ* moves according to the ALSD Hamiltonian,

(3) $$H_{\text{ALSD}}=E_{\text{ALSD}}+K+m_{\lambda }{\dot{\lambda }}^{2}/2+\text{RTln}P\left(\lambda ,T\right)$$

where *K* is the kinetic energy of the system, *m*_*λ*_ and $\dot{\lambda }$ are the fictitious mass and the velocity of the *λ*, *R* is the gas constant, *T* is the simulation temperature, and *P*(*λ*, *T*) is a canonical probability distribution at *λ*. The fictitious mass, *m*_*λ*_, was set to be the sum of the masses of the region *A*, as described in the original ALSD paper [57]. When λ=1, the *E*_ALSD_ is the same as the normal potential energy *E*. When 0<λ<1, the interactions involving the region *A* are weakened and the conformational changes in the region *A* tempt to be enhanced. The last term, $\text{RTln}P\left(\lambda ,T\right)$, is an umbrella potential to regulate the movement of the *λ*. If the priori, unknown function $P\left(\lambda ,T\right)$ is accurately estimated, the *λ* does a random walk in the predefined *λ* range (0.6<λ<1.03 in this work) and the simulation realizes both enhancing of conformational change and sampling of stable conformation for the region *A* at once. In practice, iterative simulation runs are carried out to estimate the $P\left(\lambda ,T\right)$ before a productive run. Finally, the productive run yields various conformations with changing λ. The appearance probability of each conformation was reweighted with the ALSD weighting factor according to the reweighting scheme [57] to reconstruct canonical ensembles at λ=1.

### 2.2 Zero‐Multipole Summation Method (ZMM) Including Zero‐Dipole Summation Method (ZDM)

Conventional cutoff‐used calculation, in which the interactions with all atoms within a finite small cutoff radius *r*_c_ for any atom *i*, result in large electrostatic energy errors due to artificially generated non‐electro‐neutral states. Instead, ZMM precisely calculates electrostatic interactions by considering only interactions with a subset (zero multipole subset) of atoms that satisfy the electrostatic neutral state from charge zero (*l* = 0) to the *l*‐th multipole zero within the cutoff radius *r*_c_ (zero *l*‐th moment condition). However, since ZMM is computationally expensive to extract the subset in practice, Fukuda et al. formulated the equation to approximate the electrostatic interaction from the subset by adding correction terms to the simple pairwise sum function that is used in the conventional cutoff calculation. The electrostatic interaction potential is represented as:

(4) $$\frac{1}{4\pi \epsilon_{0}}\sum_{i\in N}\sum_{\begin{array}{c} j\in N\\ j>i \end{array}}\frac{q_{i}q_{j}}{r_{\mathrm{ij}}}\approx \frac{1}{4\pi \epsilon_{0}}\left\{\sum_{i\in N}\sum_{\begin{array}{c} j>i\\ r_{\mathrm{ij}}<r_{c} \end{array}}q_{i}q_{j}\left[\frac{1}{r_{\mathrm{ij}}}-\sum_{m=0}^{l}a_{m}^{\left(l\right)}r_{\mathrm{ij}}^{2m}\right]-\frac{a_{0}^{\left(l\right)}}{2}\sum_{i}q_{i}^{2}\right\}$$

where *N* is the number of atoms in the system, and *a*_*m*_ are constants depending on *l* and *r*_c_ [68, 69, 70]. ZMM considers that a zero multiple subset consisting of the majority of atoms in the cutoff sphere satisfies zero *l*‐th moment condition, while the rest subset (excess subset) consisting of a few atoms in the thin shell near the cutoff surface disturbs the neutrality. Electrostatic interaction energy from the zero multipole subset for atom *i* can be calculated by subtracting the excess subset‐derived energy from the conventional cutoff energy expressed in the first term of Equation (4). The energy from the excess subset is approximated by a polynomial as expressed in the second term. The last term is the “self” term, which also appears in the Ewald method. For the detailed derivation, see the previous studies [68, 69, 70]. Note that the form of Equation (4) corresponds to the equation for the damping factor α=0 in the original ZMM papers. ZMM with *l* = 0, i.e., zero charge condition, is equivalent to the Wolf method [88, 89], and ZMM with *l* = 1 is zero‐dipole summation method (ZDM) [90, 91], which was used in this work.

## 3 MD Simulation Models and Procedures

Two small proteins with different secondary structures, a chignolin (PDB ID: 1UAO) [94] constituted with a β‐sheet and a trp‐cage (PDB ID: 1L2Y) [95] with an α‐helix, were selected as test systems. For each protein, the simulation system was set up as follows. As for the initial conformation, a fully elongated conformation was created using the *Z*‐matrix of amino acids. The elongated protein was relaxed with an MD simulation at a constant energy scaling factor *λ* = 0.6 (constant lambda MD, CLMD) at 300 K in vacuum with ZDM and the dielectric constant = 80 for 11 ns. The *λ* = 0.6 corresponds to 833 K (~300 K/0.6^2^) in REST2. The protein was immersed in a water sphere: the system center was the geometric center of mass of the protein, the radii were 19 and 30 Å for chignolin and trp‐cage, respectively, and the water molecules were equilibrated at 300 K and 1 g/cc in advance. Water molecules overlapping the protein were removed. Some water molecules were replaced with Na^+^ and Cl^−^ ions to neutralize the net charge and to bring the ion concentration close to physiological (0.153 M). Finally, the systems consisted of 2842 atoms (138 atoms for the protein, 1 Cl^−^ ions, 3 Na^+^ ions, 2700 atoms for water) for chignolin and 11,363 atoms (304 atoms for the protein, 10 Cl^−^ ions, 9 Na^+^ ions, 11,034 atoms for water) for trp‐cage. To compare the results obtained by the two different electrostatic energy calculation methods, two sets of simulations were performed for all subsequent simulation processes for ZDM and CMM, respectively. After energy minimization calculation, 60 CLMD simulation runs at *λ* = 0.6 were performed with different initial velocities of atoms for 1 ns. In these simulations, position constraints were applied to all heavy atoms (except hydrogens) to prevent chemically unrealistic distortions in bond lengths, angles, or dihedral angles in the early stages of the simulations. The system was then relaxed in subsequent 8 ns CLMD simulations without the position constraints. The CLMD simulations with low *λ* enhanced conformational change of the protein to generate different initial conformations for the following ALSD simulations. To speed up the sampling, the ALSD simulation was combined with a parallel computing method, trivial trajectory parallelization (TTP) [96]. The TTP samples a conformational ensemble with *N* (= 60, in this work) multiple, independent simulations starting from different initial conformations. The final conformations obtained from the CLMD simulations were used as the initial conformations for ALSD. For chignolin, iterative runs to estimate the $P\left(\lambda ,T\right)$ were carried out for 24 cycles, for the total simulation time 40ns×60runs=2,400ns and the following productive runs to sample a conformational ensemble for analyses were performed for 60ns×60runs=3,600ns. For trp‐cage, a larger than chignolin, 25 cycles of iterative runs for 70ns×60runs=4,200ns were performed and productive runs for 210ns×60runs=12,600ns were performed. Snapshots were output every 5 ps and the total number of output conformations in the productive runs were 720,000 and 2,520,000 for chignolin and trp‐cage, respectively.

As reference simulation, constant temperature MD (canonical MD) simulations at 300 K were also performed. The system setup was the same as for the aforementioned ALSD simulations except for the below. For each system, the Model 1 in the PDB was used as the initial structure. The difference in the shape of the initial structures caused a slight difference in the number of water atoms (2718 atoms for chignolin and 11,052 atoms for trp‐cage). After energy minimization calculation, 60 MD simulation runs with position constraints on heavy atoms were carried out with different initial velocities, followed by subsequent simulations without positional constraints for 60ns×60runs=3,600ns (chignolin) and 90ns×60runs=5400ns (trp‐cage), respectively. For the analyses, the first 10 ns of each run was removed as a buffer period. As with the ALSD simulations, snapshots of the MD simulations were saved every 5 ps and the total number of output conformations used in analyses were 600,000 and 960,000 for chignolin and trp‐cage, respectively.

As a more highly polarized system, we performed conformational sampling of a poly‐lysine heptapeptide, Ace‐(Lys)_6_‐Nme, capped by acetyl (Ace) and N‐methyl groups to cancel the N‐ and C‐terminal charges, respectively. The simulation system was prepared in the same manner as those for chignolin and trp‐cage. Only the differences specific to this system are described below. Unlike typical globular proteins, the peptide tends to adopt more extended conformations due to strong electrostatic repulsion among the positively charged lysine side chains. To accommodate such extended structures during solvation, the radius of the surrounding water sphere was set to a relatively large value of 28 Å. The final system contained 9211 atoms (144 atoms from the peptide, 11 Cl^−^ ions, 5 Na^+^ ions, and 9051 atoms from water molecules). Iterative runs were carried out for 24 cycles, for the total simulation time 40 ns × 60 runs = 2400 ns and the following productive runs were performed for 90 ns × 60 runs = 5400 ns.

We used j_presto [35], an MD simulation program implemented ALSD and ZDM, and force field parameters taken from Amber‐based hybrid force field (*ω* = 0.75) [97], TIP3P [98], and Joung‐Cheatham [99] for the proteins, water molecules, and ions, respectively. During the MD simulations, a harmonic potential to avoid evaporation of water molecules from the water sphere boundary was applied to the center of mass of water only when it was outside the boundary. A time step of simulation was 2 fs. Covalent bonds involving hydrogen atoms were constrained by the SHAKE algorithm [100]. Simulation temperature was set to 300 K and controlled by the constant‐temperature method [101]. To investigate the effects of different electrostatic energy calculation methods, we used the zero‐dipole summation method (ZDM), which assumes electrostatic neutrality of charges and dipoles, as one of ZMMs, and the cell multipole method (CMM) as a conventional method. CMM is applicable even when the total charge of the system is non‐zero, unlike Ewald‐based methods that assume total net charge neutrality. We used a 12 Å cutoff distance for both electrostatic and vdW energy calculations in the ZDM simulations. In the CMM simulations, a 12 Å cutoff distance was applied only to vdW interactions, as electrostatic interactions were computed approximately without using a cutoff. For the poly‐lysine system, additional ZDM simulations with a 16 Å cutoff distance were also performed to evaluate the cutoff‐distance dependence of long‐range electrostatic repulsion. The neighbor list was updated every 5 and 10 steps for vacuum and solvated systems, respectively.

## 4 Free‐Energy Landscapes on the Principal Component Subspace

Free‐energy landscapes (FELs) were constructed with principal component analysis (PCA). For chignolin system, each conformation was expressed as a 28‐dimensional vector $q=\left[q_{1},q_{2},\cdots ,q_{28}\right]$, where *d*_ij_ is the minimum distance among heavy atoms in the *i*‐th residue and those in the *j*‐th residue when $\left|i-j\right|>2$ and $q_{1}=d_{1,4}$, $q_{2}=d_{1,5}$, ⋯, $q_{28}=d_{7,10}$. Then a weighted variance–covariance matrix was calculated as

(5) $$\begin{array}{c} M_{\mathrm{ij}}=\left\langle w∙q_{i}q_{j}\right\rangle -\left\langle w∙q_{i}\right\rangle \left\langle w∙q_{j}\right\rangle \end{array}$$

where $\left\langle \cdots \right\rangle$ means the average of the two ensembles from ALSD simulations with ZDM or CMM, and *w* is the ALSD weighting factor. By diagonalizing the matrix *M*, we obtained 28 eigenvectors ***v***_k_ and corresponding eigenvalues *e*_k_, where *k* denotes the *k*‐th eigenvector and eigenvalue, respectively, and rearranged the eigenvalues in descending order. A two‐dimensional principal component (2D PC) subspace was constructed using the two eigenvectors, ***v***_1_ and ***v***_2_. A conformation ***q*´** was projected at the 2D coordinates [*c*_1_, *c*_2_] of ***q*´**: $c_{i}=v_{i}∙\left(q^{\prime }-\left\langle q\right\rangle \right)$ with a weight *w*. The probability distributions *P*(*c*_1_, *c*_2_) in the 2D PC subspace were converted to the potential of mean forces, $\mathrm{PMF}\left(c_{1},c_{2}\right)=-\text{RTln}P\left(c_{1},c_{2}\right)$, as FELs. Similarly, trp‐cage and poly‐lysine heptapeptide conformations were expressed as 153‐ and 15‐dimensional vectors, respectively, and projected in their 2D PC subspaces.

## 5 Results and Discussion

**FIGURE 1:**
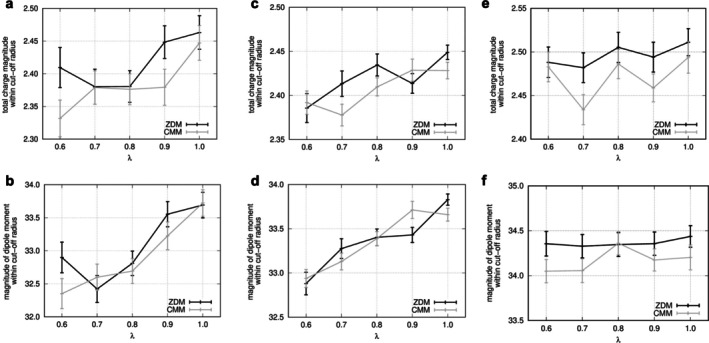
Local electrostatic environment around solute atoms, where atomic charges are scaled by *λ* during ALSD simulation, within a 12 Å cutoff radius. Average magnitudes of total charge (a, c, e) and dipole moment (b, d, f) are shown for chignolin (a, b), trp‐cage (c, d) and poly‐lysine heptapeptide (e, f). Total charge magnitude represents the absolute sum of atomic charges within the cutoff radius. Error bars indicate standard errors, calculated based on the number of data points within each *λ* bin.

To assess potential differences in the local electrostatic environment caused by the distinct methods used in ZDM and CMM, we analyzed the magnitudes of average monopole (absolute value of total charges) and dipole moments scaled by *λ* within a 12 Å cutoff radius around solute atoms enhanced conformational changes with ALSD. Figure 1 shows the average values for each *λ* and their standard errors. As *λ* decreases, the charges of solute atoms are scaled down during the simulations, which causes the reduction of both monopole and dipole. The results showed no significant differences (exceeding two standard errors) in either the monopole or dipole between ZDM and CMM across *λ* values, indicating that the local electrostatic environment around solute atoms, despite the methodological differences, remains sufficiently similar and falls within a negligible range of variation.

**FIGURE 2:**
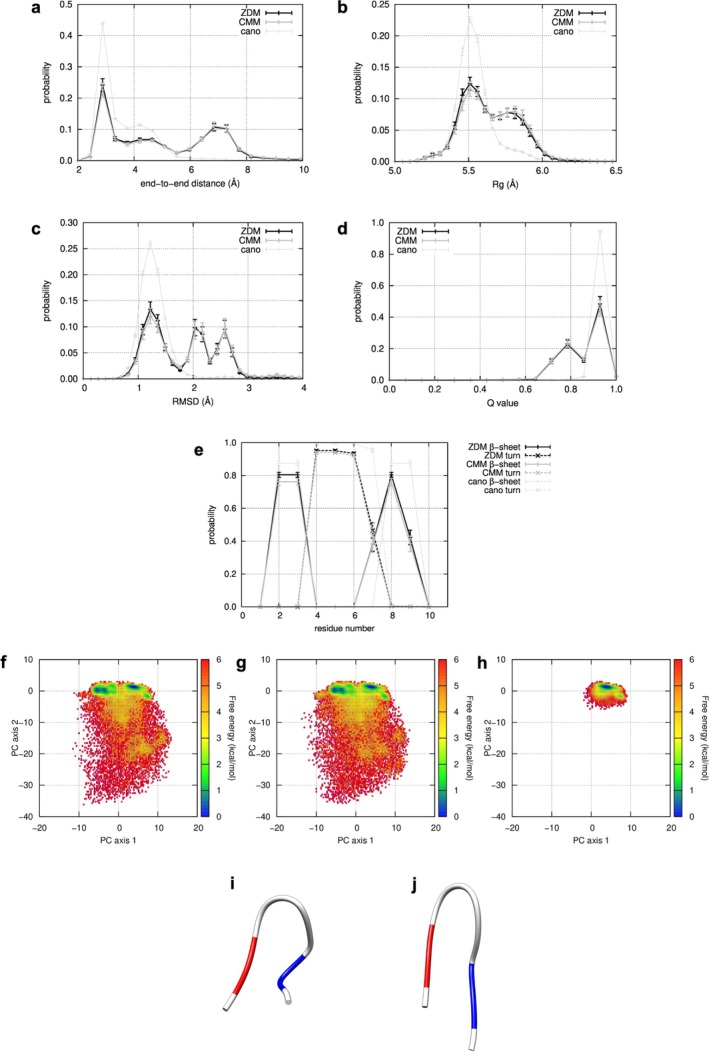
Probability distribution on reaction coordinates, the end‐to‐end distance (a), the *R*_*g*_ (b), the RMSD (c), and the *Q* value (d). Distributions obtained from ALSD with ZDM, with CMM, and canonical MD are represented by black line, gray line, and gray thin line, respectively. Reproduction rates of secondary structure at each residue (e). The probability of β‐strand and hydrogen bonded turn are represented as solid and dotted lines, respectively. Free‐energy landscapes (FELs) from ALSD with ZDM (f), with CMM (g), and canonical MD (h). Each structure sampled from simulations was represented as a vector based on distances between residues and projected onto a two‐dimensional plane consisting of the first and second principal component axes (PC axis 1 and 2) obtained by principal component analysis. The minimum RMSD structure obtained from ALSD with ZDM (i). The structure belongs to the first cluster at around [4, 2] (*RMSD* = 0.41 Å, *Q* = 1.00, coordinates on the PC subspace = [5.02, −0.39]). The two regions that form β‐strand in the native structure, residues 2–3 and 8–9, are colored red and blue, respectively. A misfolded structure located in the second cluster around [−5, 0] (j) (*RMSD* = 2.40 Å, *Q* = 0.79, coordinates on the PC subspace = [−5.00, 0.00]). The residue pairs forming β‐sheet are shifted by one residue relative to those in the native structure.

To examine if ZDM and CMM methods give significantly different conformational ensembles, we compared their probability distributions for several reaction coordinates. First, we show the distributions of the end‐to‐end distance in Figure 2a. The end‐to‐end distance was calculated as the distance between the first residue's nitrogen atom and the last residue's oxygen atom. The averages of the distributions and their standard errors were calculated from 60 simulation runs performed in parallel with TTP, considering the ALSD weighting factors. The distributions were statistically consistent within the standard errors and were not significantly different (*p* < 0.05, two‐tailed), except for the distances of more than > 9.0 Å, in which the populations were considerably low. Both distributions exhibited a sharp maximum peak at 2.9 Å and a gentle second maximum peak at around 7.0 Å. This maximum position aligned with that obtained from the canonical MD simulations of the native structure at 300 K. In canonical MD simulations, proteins undergo thermal oscillation around their native structures without significant conformational changes like those in ALSD. Consequently, these results demonstrate that ALSD accurately reproduces the most frequent peak, irrespective of using ZDM and CMM methods.

We then compared probability distributions on the radius of gyration (*R*_*g*_). The *R*_*g*_ is a measure of the radius of a molecule, assuming that the molecule is spherical, and is calculated by the following formula $R_{g}=\sqrt{\sum_{i=1}^{N}r_{i}^{2}/N}$, where *N* is the number of heavy atoms in the protein and *r*_*i*_ is the distance from atom *i* to the geometric center of the protein. As shown in Figure 2b, the distributions also agreed well and were not considerably different except for distances more than 6.2 Å. Both distributions commonly had a sharp maximum peak at 5.5 Å, as shown in the canonical MD simulations, and a gentle second maximum peak at around 5.8 Å.

Next, we compared the probability distributions of the root mean square deviation (RMSD) for *C*_*α*_ atoms and the reproducibility of inter‐residue contacts (*Q*‐value), which is a measure of the similarity of conformations to the native structure (PDB ID: 1UAO model 1). The *Q*‐value was calculated based on the residue pairs three residues apart in sequence and located within 6 Å in the native structure, defined as the Native Contact Pair (NCP, the total is 14 pairs in the chignolin). During simulations, when the distance was within 7 Å, the NCP was judged to be reproduced. The *Q*‐value, defined as the ratio of the number of NCPs reproduced to the total number of NCPs, was calculated for each simulated structure. The *Q*‐value ranges from 0 to 1. The value closer to 1 indicates better reproducibility of the native structure. The probability distributions on the RMSD and the *Q*‐value are shown in Figure 2c,d, respectively. These results also show no significant differences between the two ensembles, and the positions of their maximum peaks coincided with those from the canonical MD simulations. In addition to the maximum peaks, the RMSD distributions obtained by ZDM and CMM show two additional peaks at around 2.0 and 2.6, respectively. Similarly, the *Q*‐value distribution exhibited a smaller peak below 0.8. These peaks correspond to known misfolded conformations (see below).

The reproduction rates of the secondary structure were also examined. The secondary structures were calculated using the DSSP program [102] (β‐strand is E and hydrogen bonded turn is S in DSSP determination, respectively). The reproduction rates show no significant differences between the two ensembles in Figure 2e. However, they differ from the canonical MD simulations at the 7th and 9th residues. The turn conformation at the 7th residue is partially replaced by the β‐strand, and the β‐strand at the 9th residue is partially disrupted in the simulated structures. In summary, the results indicate no significant differences between the ensembles obtained with ZDM and CMM for the five measures, indicating that they correctly reproduced the maximum peak locations in the probability distributions obtained from canonical MD simulations. However, some small peaks were observed in ZDM and CMM, but not in the canonical MD.

To investigate further the differences from the canonical MD simulations, we constructed 2D‐FELs for the two largest principal components. The FELs from ZDM and CMM are shown in Figure 2f,g, respectively. These FELs are similar and hold two common, large conformational clusters around [4, 2] and [−5, 0]. The structures (Figure 2i) in the cluster at [4, 2] were close to those from the canonical MD simulations. In contrast, the structure in the cluster at [−5, 0] took different conformations (Figure 2j). In the cluster at [−5, 0], the residue pairs forming the hydrogen bonds for β‐sheet (residue pairs of 2 and 8 and 3 and 7) were shifted by one residue relative to those in the native structure (residue pairs 2 and 9 and 3 and 8). In a previous simulation study [103], these conformations have been reported as misfolded conformations of chignolin [103]. In these structures, the turn at the 7th residue in the native structure was replaced by the β‐strand, and the β‐strand at the 9th residue was missing. We conclude that the partial differences in the reproduction rate of secondary structures come from the cluster at [−5, 0].

**FIGURE 3:**
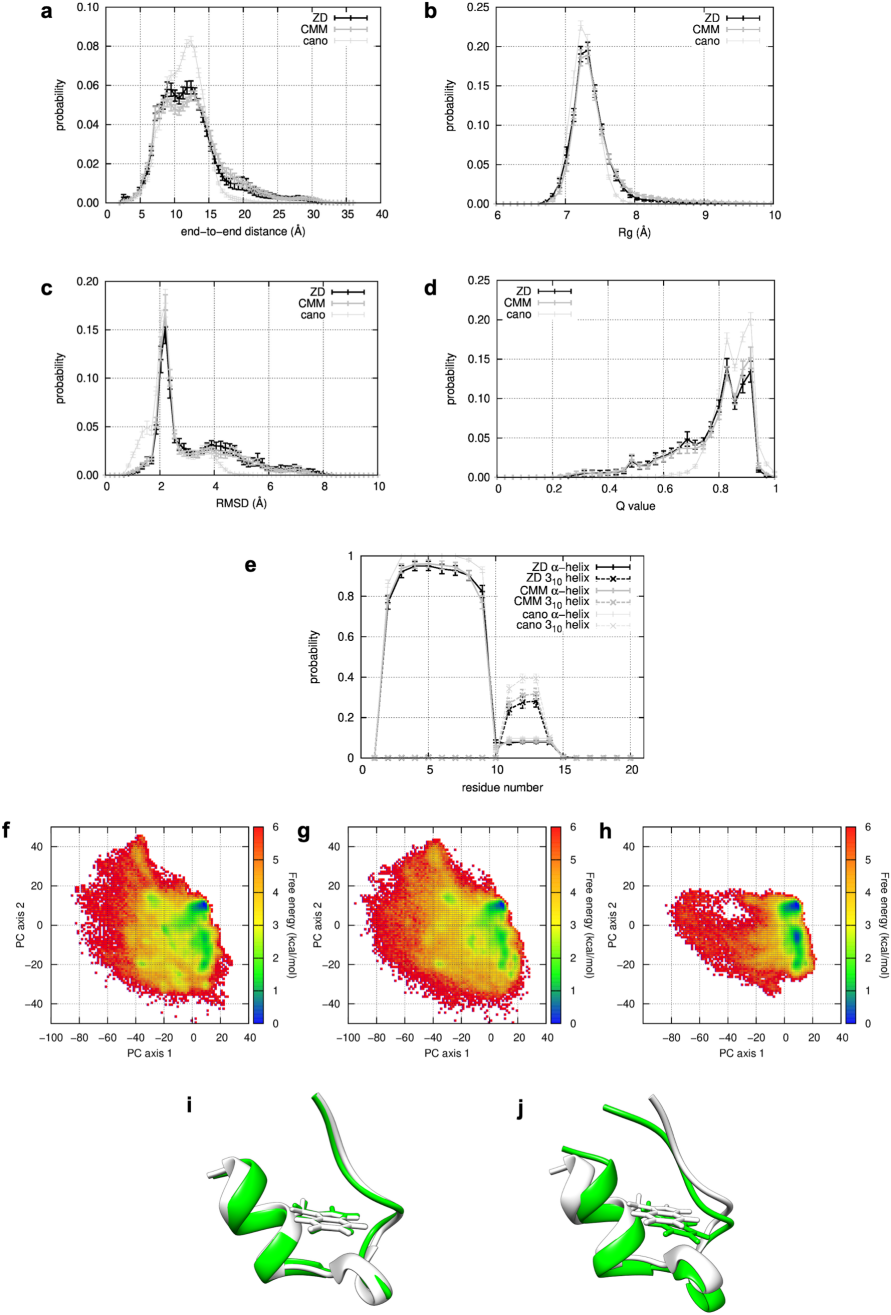
Probability distribution on reaction coordinates, the end‐to‐end distance (a), the *R*_*g*_ (b), the RMSD (c), and the *Q* value (d). Reproduction rates of secondary structure at each residue (e). The probability of α‐helix and 3_10_ helix are represented as solid and dotted lines, respectively. Free‐energy landscapes (FELs) from ALSD with ZDM (f), with CMM (g), and canonical MD (h). The minimum RMSD structure obtained from ALSD with ZDM (i, green) and the native structure (i, white). The structure is located in the cluster around [10, −5] (*RMSD* = 0.48 Å, *Q* = 1.00, coordinates on the PC subspace = [6.22, −0.49]). A structure located in a cluster around [10, 10] obtained from ALSD with ZDM (j, green) (*RMSD* = 2.32 Å, *Q* = 0.89, coordinates on the PC subspace = [10.00, 10.00]) and the native structure (j, white).

We then applied a similar analysis for the trp‐cage system. The RMSD and the *Q* value were used as measures of the similarity to the native structure (PDB ID: 1L2Y model 1). As in the case of chignolin, no significant differences in distributions on the end‐to‐end distance (Figure 3a), the *R*_*g*_ (Figure 3b), the RMSD (Figure 3c), and the *Q* value (Figure 3d) were detected. The distributions have the maximum peak that commonly appears in the distribution of the canonical MD. The reproduction rates of the secondary structure to the native conformation were calculated for the two types of secondary structures, α‐helix and 3_10_ helix (H and G in DSSP determination, respectively). The reproducibility of the α‐helix was high (approximately 90%) in the residues 2–7, corresponding to the region forming the α‐helix in the native structure, as shown in Figure 3e.

The 2D‐FELs obtained from ALSD with ZDM and CMM are shown in Figure 3f,g, respectively. These FELs exhibit striking similarities, with the most prominent conformational cluster consistently emerging near [10, 10]. The structures in the cluster corresponded to the most stable conformation in the FEL from the canonical MD simulations (Figure 3h). However, the FEL of the canonical MD differed from ALSD‐derived FELs in that a cluster near [10, −5], where structures with small RMSD (about 0–2 Å, Figure 3i), takes a lower free energy value. The structural ensemble obtained from the canonical MD simulations depends on the initial structure because the conformation changes much more slowly than ALSD, causing the overestimation of the cluster. As shown in Figure 3c, the canonical distribution had a small peak at RMSD ~1.5 Å, corresponding to the structures in the cluster at [10, −5]. However, the maximum peak emerged around 2.2 Å, corresponding to the conformations of the cluster at [10, 10]. These results indicate that the initial structure (1L2Y model 1 determined by the NMR experiment) may not be the most stable. The structure with a slight conformational change from the initial one will be the most stable, which belongs to a cluster at [10, 10], with the RMSD of 2–2.5 Å and the *Q*‐value of 0.8–0.9 (Figure 3j).

**FIGURE 4:**
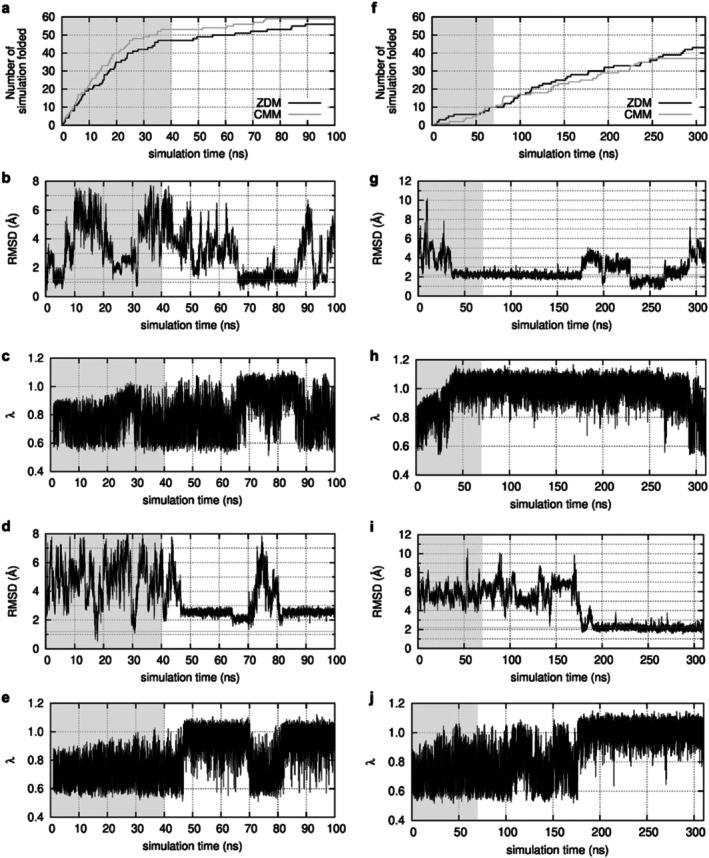
Elapsed time of runs to reach the native structure for chignolin from the extended, initial conformations (a). The gray background indicates the period for ALSD iterative runs. Time evolution of the RMSD (b) and the *λ* (c) of the fastest folding run. The gray horizontal line is the threshold (*RMSD* = 1.2 Å) for whether chignolin is folded to the native structure. Time evolution of the RMSD (d) and the *λ* (e) of the 30th folding run, the middle of all 60 runs. Elapsed time to reach the native structure for trp‐cage from the extended, initial conformations (f). Time evolution of the RMSD (g) and the *λ* (h) of the fastest folding run with the threshold (*RMSD* = 2.2 Å). Time evolution of the RMSD (i) and the *λ* (j) of the 30th folding run.

We evaluated the simulation time required for ALSD to reach the native structures starting from fully unfolded structures and compared the folding speeds between ZDM and CMM. Figure 4a shows the number of MD runs that reached the native structure at least once out of 60 runs for chignolin. We regarded each run as folded into the native structure when the RMSD reached 1.2 Å or less, which corresponds to the maximum peak in the RMSD distribution (Figure 2c). The fastest folding runs to the native structure took 0.295 ns for ZDM and 0.105 ns for CMM. The 30th folding run (the median of all 60 runs) took 17.025 ns for ZDM and 13.615 ns for CMM. For trp‐cage, the fastest folding runs using ZDM and CMM were 4.450 and 3.180 ns, while the 30th folding runs were 177.525 ns and 211.375 ns with RMSD criterion of 2.2 Å for the folding (Figure 4f). Figure 4b–e,g–j show the changes in RMSD and *λ* for the fastest and the 30th folding runs for chignolin and trp‐cage, respectively. The folding time was comparable in ZDM and CMM, showing no significant differences. Therefore, ALSD combined with ZDM or CMM did not differ significantly in the explored conformational ensembles and the sampling speed for systems relatively neutral in charge.

**FIGURE 5:**
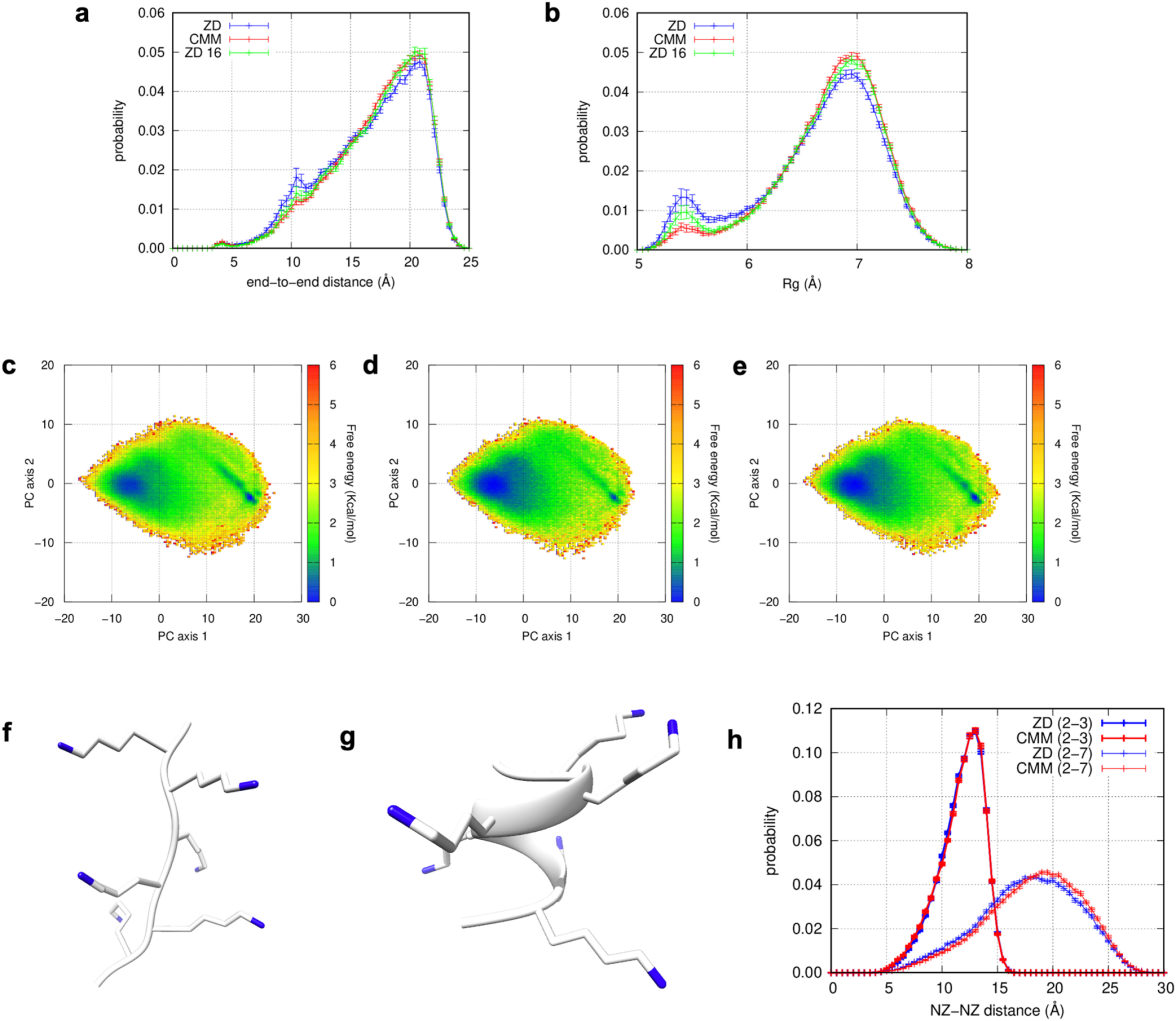
Probability distribution on the end‐to‐end distance (a), the *R*_*g*_ (b). Blue: ZDM; Red: CMM; Green: ZDM with 16 Å cutoff (labeled as “ZD 16”). Free‐energy landscapes (FELs) from ALSD with ZDM (c), with CMM (d), and with 16 Å cutoff ZDM (e). Examples of extended (f) and helical (g) structures at [−7, −1] and [−19, −2.5], respectively, on the FEL obtained from ALSD with ZDM. NZ atoms are colored blue. Probability distributions on the distance between NZ atoms of lysine residues (h). Thick red and blue lines represent tthe NZ‐NZ distance distribution between adjacent lysine residues (2Lys‐3Lys). Thin red and blue lines correspond to the NZ‐NZ distances between the most distant lysine residues in the protein (2Lys‐7Lys). Thin and thick lines distinguish residue pairs with different sequence separations, highlighting the effect of chain connectivity on spatial arrangement.

Next, to investigate whether similar trends would hold for systems with higher polarity, we carried out conformational sampling for a more highly polarized model system: a poly‐lysine heptapeptide, consisting solely of positively charged lysine residues. Unlike chignolin and trp‐cage, this peptide does not have an experimentally determined native structure. Accordingly, no analyses that require the native structure as reference—such as RMSD, *Q*‐value, and folding time—were performed for this system. We found that significant differences in the end‐to‐end distance and the *R*_*g*_ distributions (Figure 5a,b) but not in the magnitudes of the average monopole and dipole moments (Figure 1e,f). As illustrated by the free energy landscapes (Figure 5c,d), this peptide predominantly adopts extended conformations ([−7, −1] in Figure 5f) due to electrostatic repulsion between the positively charged lysine side chains, although it occasionally forms α‐helical structures ([19, −2.5] in Figure 5g) with low probability. Both conformations are found in simulations using either CMM or ZDM; however, the population of α‐helical structures in the ZDM is higher compared with CMM. In the distributions of the end‐to‐end distance and *R*_*g*_ for ZDM, distinct peaks corresponding to α‐helical structures are observed at 11 and 5.4 Å, respectively. In contrast, such peaks are absent or very small in the distributions of CMM. Instead, CMM exhibits higher probabilities in regions corresponding to extended conformations, around 21 and 7 Å, respectively.

To better understand why ZDM tends to produce a higher population of α‐helical structures compared to CMM, we considered the physical requirements for helix formation in the poly‐lysine peptide. Due to the strong electrostatic repulsion between the positively charged lysine side chains, poly‐lysine generally adopts extended conformations. Transitioning from an extended to a helical conformation requires overcoming this repulsive energy barrier. The higher frequency of α‐helical structures in ZDM simulations suggests an underestimate of this repulsive energy in ZDM. To examine this possibility, we analyzed the distributions of distances between the terminal NZ atoms of adjacent lysine side chains (2LYS and 3LYS; Figure 5h). The NZ‐NZ distances ranged from approximately 5–16 Å, with a peak around 13 Å. In ZDM, electrostatic interactions were evaluated based on local electrostatic neutrality within a finite cutoff radius, which was 12 Å in our simulations. This means that repulsive interactions between NZ atoms separated by ~13 Å—beyond the cutoff—are explicitly included in CMM, but not directly accounted for in ZDM. As a result, NZ‐NZ interactions beyond the cutoff radius were neglected in ZDM, causing an underestimate of the electrostatic repulsion compared to CMM.

To determine whether this cutoff treatment in ZDM accounts for the discrepancy in the ensembles, we extended the cutoff radius to 16 Å, which ensures that electrostatic repulsion between all adjacent lysine side chains—separated by up to 16 Å—is explicitly included. As a result, the population of α‐helical structures decreased, and the distributions of end‐to‐end distance and *R*_*g*_, and the free energy landscape, became more similar to those obtained using CMM (Figure 5a,b,e). Although the *R*_*g*_ distribution still shows a subtle peak in the helical region, no statistically significant differences were detected between the two methods. One possible explanation is that, even with the extended cutoff, electrostatic repulsion between non‐adjacent lysine residues may still not be fully captured (Figure 5h). These results indicate that the ensemble differences observed between CMM and ZDM are not due to the combination of ALSD with ZDM, but rather stem from a previously unrecognized limitation intrinsic to ZDM itself—namely, its sensitivity to the choice of cutoff radius in the systems in which long‐range repulsion is dominated. This finding highlights the importance of carefully selecting the cutoff radius based on the nature of electrostatic interactions in the target system.

It is difficult to mathematically prove that the conformational ensembles and statistical physical quantities obtained from the combination of GEPS and ZMM are essentially equivalent to those obtained using conventional electrostatic interaction methods. Therefore, as an alternative to mathematical proof, we adopted an approach to obtain empirical evidence that supports the equivalence of the two approaches. We evaluated the results of two well‐studied, relatively neutral proteins, chignolin and trp‐cage, as well as a highly polarized poly‐lysine heptapeptide. For the relatively neutral proteins, the combination of ALSD with ZDM showed no significant differences in the local electrostatic environment around atoms compared to ALSD with CMM. Moreover, the resulting conformational ensembles exhibited no substantial differences in the five measures and the FELs. Both methods accurately reproduced the native conformational cluster as the most stable state, and the conformational search speed was compatible. Our results demonstrate that GEPS and ZMM can be effectively combined without introducing systematic bias, at least for small proteins (typically 10–20 residues). This conclusion is supported not only by the consistency observed in relatively neutral systems but also by the improved agreement achieved in a highly polarized poly‐lysine system with an appropriately large cutoff radius. These findings provide guidance for applying GEPS with ZMM in conformational sampling of biomolecules.

Through the analyses, we found a potential limitation inherent to ZMM itself: its sensitivity to the cutoff radius in systems where long‐range electrostatic repulsion is dominant. ZMM assumes local electrostatic neutrality and has been successfully applied to systems composed of small, mobile molecules such as ions or water. In such systems, diffusion and spatial averaging effectively prevent localized charge accumulation, automatically maintaining local neutrality. However, in chain‐like molecules such as poly‐lysine, structural constraints imposed by covalent bonds limit the spatial mobility of charges. Moreover, the positively charged side‐chain termini of lysine residues are embedded within a hydrophobic framework, making it difficult for surrounding ions and water molecules to effectively neutralize their charge. As a result, compared to ions or water systems, charge accumulation is more likely to occur at specific spatial locations, potentially violating the local electrostatic neutrality assumption of ZMM. For instance, when such localized charges lie just outside the cutoff radius, the effect is explicitly included in CMM, but not in ZMM, where interactions beyond the cutoff radius are not directly computed. Although the effect on individual structures may be small, ensemble‐level comparisons revealed statistically significant differences. The good agreement observed in the neutral protein systems can be attributed to the absence of strong charge distribution inhomogeneity. We demonstrated that increasing the ZMM cutoff radius for poly‐lysine reduced the discrepancies relative to CMM. However, this approach increases computational costs. Furthermore, the optimal cutoff radius cannot be determined in advance. These findings underscore the importance of carefully selecting the cutoff radius when applying ZMM to highly polarized biomolecular systems.

Parameter‐variable GEPSs, which adjust atomic charges during simulations, require careful consideration of electrostatic interaction calculation methods that assume electrostatic neutrality. For instance, Ewald‐based methods used to calculate electrostatic interactions—widely used in simulation studies—presume that the total charge of the system remains zero. Since GEPSs only modify the charges in the pre‐defined regions, the overall charge of the system may deviate from zero during simulations. Therefore, we employed CMM, a non‐Ewald‐based method, as the standard approach for handling electrostatic interactions. It will be important to verify whether the change variations introduced by GEPSs are compatible with the Ewald method and how they might affect the investigated conformational ensembles; however, this inquiry lies beyond the scope of the current study.

## Conflicts of Interest

The authors declare no conflicts of interest.

## Data Availability

The data that support the findings of this study are available from the corresponding author upon reasonable request.
